# Toxicity Screening of Single Dose of Inorganic and Organic Arsenics on Hematological and Serum Biochemical Parameters in Male Cynomolgus Monkeys

**DOI:** 10.5487/TR.2008.24.3.219

**Published:** 2008-09-01

**Authors:** Choong-Yong Kim, Kang-Hyun Han, Jeong-Doo Heo, EuiSik Han, YoungNa Yum, Jin-Young Lee, KyungSu Park, Ruth Im, Seong-Jin Choi, Jung-Duck Park

**Affiliations:** 110 Korea Institute of Toxicology, KRICT, P.O. Box 123, Yuseong, Daejeon, Korea; 210 National Institute of Toxicological Research, Korea; 310 Advanced Analysis Center, Korea Institute of Science and Technology, Korea; 410 Dept. Preventive Medicine, College of Medicine, Chung-Ang University, 221 Huksukdong, Dongjakgu, Seoul, 156-756 Korea

**Keywords:** Cynomolgus monkeys, Organic arsenic, Toxicity screening, Single dose

## Abstract

A screening study of the acute toxicity of organic arsenics such as arsenobetaine and arsenocholine, a product of arsenic methylation metabolite, and inorganic arsenic was carried out to examine hematological and serum biochemical parameters in cynomolgus monkeys (*Macaca fascicularis*). We found soft and liquid feces, and vomiting in all treated groups with inorganic and organic arsenics. The monkeys in inorganic arsenic-treated group showed a significant increase in vomiting frequency compared with those in three organic arsenics-treated groups. These results suggest that inorganic arsenic might be more toxic than three other organic arsenics tested. The monkeys in inorganic arsenic-treated group showed a decrease in platelet and an increase in monocyte on day 4 and the monkeys in arsenocholine-treated group showed an increase in reticulocyte percentage on day 8. The monkeys in inorganic-treated group also showed decreases in AST and ALT values and the monkeys in arsenobetaine-treated group showed a decrease in AST value and an increase in T-CHO value. However, these hematological and biochemical changes were within the physiological ranges, showing that the single dose of inorganic and organic arsenics did not affect at least hematological and serum biochemical parameters. The present study of toxicity with single dose of arsenics provides valuable indicators for longer term study of toxicity of repeated doses of arsenics in primates.

## INTRODUCTION

Arsenic (As) is an ubiqitous element found in food and water, and is a potent toxicant that may exist in several oxidation states. Although a certain food, such as marine fish, contains substantial levels of arsenic forms (e.g., arsenobetaine and arsenocholine), the toxicity is relatively low compared to the toxicity in inorganic aresenics (Mandal and Suzuki, 2002; Vahter, 2000). Arsenocholine is detected at low levels, about 0.3% total arsenics, from shrimp and conch, and it is thought to be a candidate of arsenobetaine precursor in the marine food chain. Arsenobetaine is a major organic compound in marine animals and a final metabolite in the arsenic cycle of marine ecosystem. A previous study reported the *in vitro* cytotoxicity of arsenocholine in murine immune effector cells (Sakurai, 2002).

Trivalent arsenic, an arsenite form, has a high affinity for protein sulfhydryl groups, but it seems to be selective in reacting with closely spaced dithiols which are common in DNA-binding proteins, transcription factors, and DNA-repair enzymes. Exposure of inorganic arsenics to human has been associated with a variety of effects on health, including increases in cardiovascular disease, neurological defects, and neoplasias of the skin, liver, kidney, and bladder (Guha Mazumder *et al.*, 1998). It had been widely accepted that the methylation of inorganic arsenic leads a detoxification, like as methylated arsenics were reported to be less acutely toxic, less reactive with tissue macromolecules, and more readily excreted than their inorganic counterparts. Compared with inorganic arsenic, methylarsonic acid (MMA) and dimethylarsinic acid (DMA) which are the final products of arsenic methylation, show a low degree of toxicity in a variety of test systems (Kreppel *et al*., 1993; Oya-Ohta *et al*., 1996; Moore *et al*., 1997; Rasmussen and Menzel, 1997; Sakurai *et al*., 1998). The methylated metabolites are also less reactive with tissue constituents than inorganic arsenic, and more readily excreted in the urine (Buchet *et al*., 1981; Vahter *et al*., 1984; Hughes and Kenyon, 1998).

While it is generally accepted that methylation of organic and inorganic arsenics is the principal detoxification pathway, recent studies have suggested that methylated metabolites may be partly responsible for the adverse effects associated with arsenic exposure (Styblo and Thomas, 1997; Styblo *et al*., 1997; Li *et al*., 1998; Lin *et al*., 1999; Vega *et al*., 2001). There is a considerable variation of sensitivity to toxicity among mammalian species (Vahter, 1994, 1999). In acute toxicity among mammalian species, humans seem to be more sensitive than experimental animals. It has been also known that among non-human primates, there is a difference in activity of methyltransferase to convert non-methylated arsenic to methylated form. For instance, the marmoset monkey and the chimpanzee lack the ability to methylate arsenic, while the cynomolgus and rhesus monkeys can methylate it (Wildfang *et al*., 2001; Vahter, 2000). However, toxic effects of inorganic arsenic and organic arsenic have not been intensively investigated in cynomolgus monkeys which are able to methylate arsenics.

In the present study, we investigated the toxicity of organic arsenics (arsenobetaine and arsenocholine), and one of the final products of arsenic methylation (DMA), and inorganic arsenic (sodium arsenite) using hematological and serum biochemical parameters and these parameters will be indicator for the study of toxicity of the repeated dose of arsenicals in cynomolgus monkeys.

## MATERIALS AND METHODS

***Animals.*** Twelve male cynomolgus monkeys, captive bred at Guangxi Primate Center, China, were used in this study. Monkeys were obtained from Hamri Co. Ltd (Japan) at 3 to 6 years of age, and were quarantined at the non-human primate facility for 30 days before use. The monkeys were subjected to various external physical examinations, tuberculosis test, and microbiological test for *salmonella*, *shigella*, and *yersinia* examination during a quarantine period. An acclimation period (at least 4 weeks) was allowed between receipt of the animals and the start of study to make the monkeys accustomed to the laboratory environment. During this acclimation period, all animals were observed daily for any clinical signs of disease. The monkeys which have no sign of disease were selected for the study. All animals in this study were used in accordance with the principles outlined in the “Guide for the Care and Use of Laboratory Animals”, a NIH publication.

The range of body weights were from 3668 to 4410 g at the dosing initiation date. The animal room was maintained at a temperature of 23 ± 3$^{o}$C, relative humidity of 55 ± 10%, air ventilation of 10 to 20 times/hour and a light intensity of 150 to 300 Lux with a 12 hour light/dark cycle. Throughout the study, the monkeys were housed individually in stainless steel wire cage (543 W × 715 L × 818 H mm), and fed a standard monkey diet (Oriental Yeast Co., Tokyo, Japan). The UV-irradiated and filtered municipal tap water was provided to animals *ad libitum.*

***Test article and dose selection.*** Sodium cacocylate trihydrate (DMA) and sodium arsenite were purchased from Sigma-Aldrich (St. Louis, MO, USA). Arsenobetaine and arsenocholine were purchased from Tri Chemical Lab. Inc. (Uenohara-shi, Yamanashi, Japan). Chemicals were dissolved in distilled water and were given to monkeys with two As doses (low and high doses). For dose selection, the minimal risk levels (MRL, 0.005 mg/kg/day) in human exposure was considered, and it was converted by a conversion factor of 3 in case of monkey from body surface area method (Gad and Chengelis, 1998).

***Animal group and general procedure.*** In the present study, twelve monkeys were devided to four groups (3 animals/group); one inorganic arsenic-treated group and three organic arsenic-treated groups.Sodiun aresenite was used in inorganic arsenic-treated group. Aresenocholine, aresenbetaine, and DMA were used in organic arsenic-treated gourps. The doses of sodium arsenite were 2.60 mg/kg for low-dose and 26.01 mg/kg for high-dose.The doses for arsenobetaine were 3.56 mg/kg for low-dose and 71.30 mg/kg for high-dose.arsenocholine (low-dose: 4.90 mg/kg, high-dose: 98.10 mg/kg) and DMA (low-dose: 4.28 mg/kg, high-dose: 82.70 mg/kg) These doses were selected using the Path/Tox system (Xybion Medical Systems Co., USA) based on body weight stratification before the pretreatment period.

The chemicals were given to the animals by oral administration with escalated dosing for low and high doses. The high dose groups were given on 19 days after the first dosing which was low dose on day 1. The chemicals of low-dose and high-dose were administered one time for each dose during the study period.

Each monkey was identified by tattoo on femoral region and each cage was identified by the cage card during a quarantine period. During 33-day treatment period, the chemicals were orally administered to the animals. Chemicals were dosed to the animals at a volume of 5 ml/kg. The blood samples were obtained from the cephalic vein of cynomolgus monkeys. The experimental protocol for animal use in this study was reviewed and approved by the Institutional Animal Care and Use Committee (IACUC) of KIT (Certification acquisition of Association for Assessment and Accreditation of Laboratory Animal Care (AAALAC) International in 1998).

***Clinical observations, body weight and food consumption.*** Animals were monitored once a day during the pretreatment period. During the treatment period, monkeys were monitored before and after dosing. If there were clinical signs including mortality, morbidity, general appearance, and behavior changes, those were recorded with date, time of finding, and duration. Body weights were recorded during the pretreatment and treatment period of day 1 and 19. Food consumption was recorded during the pre-treatment and treatment period of day 6, 15, 22 and 29.

***Hematological and serum biochemical assays.*** Blood samples were collected for hematological examination at day -6, 2, 4, and 8 after starting low- and high-doses administration from the monkey's cephalic vein in the tubes containing EDTA-2K (Table 1). Hematological items were measured automatically using a hematological autoanalyzer (ADVIA120 Hematology System Bayer, U.S.A).

For serum biochemical examination, blood samples were collected from the monkey's cephalic vein at day 4 after starting low and high-doses administration, allowed to clot and centrifuged at 3,000 rpm for 10 minutes to separate serum (Table 1). The sera were stored in the -80°C freezer before they were analyzed. Serum biochemical items were measured by an autoanalyzer (TBA 200FR Neo, Toshiba, Japan).

***Statistical analysis.*** The data from hematological and serum biochemical parameters was analyzed for homogeneity of variance using Bartlett's test. Homogeneous data was analyzed using the Analysis of Variance and the significance of inter-group differences was analyzed using Dunnett's t test. Nonhomogeneous data was analyzed using the Kruskal-Wallis H test and the significance of inter-group differences was analyzed using Dunn's Rank Sum test. Statistical analyses was

**Table 1.** Abbreviations, units and analysis methods of the items

| Items | Units | Methods |
|---|---|---|
| RBC (Red blood cell) | × $10^6$/mm$^3$ | Laser optical (Flow cytometry) |
| HGB (Hemoglobin concentration) | g/dl | Cyanmethemoglobin spectrophotometry |
| HCT (Hematocrit) | % | Calculation from MCV |
| MCV (Mean corpuscular volume) | Fl | Laser optical (Flow cytometry) |
| MCH (Mean corpuscular hemoglobin) | pg | (HGB/RBC) × 10 |
| RET (Reticulocyte count) | % | Laser optical with cytochemical reaction |
| RDW (Red cell distribution width) | % | Laser optical flow cytometry |
| Platelet | × $10^3$/mm$^3$ | Laser optical Flow cytometry |
| WBC (White blood cell) | × $10^3$/mm$^3$ | Laser optical with cytochemical reaction |
| Differential leucocyte count | % | Perox optical with chemical reaction |
| AST (Aspartate aminotransferase) | IU/l | GSCC(DGKC), Karman, JSCC |
| ALT (Alanine aminotransferase) | IU/l | GSCC(DGKC), Karman, JSCC |
| ALP (Alkaline phosphatase) | IU/l | P-NPP, GSCC, Bessey-Lpwry |
| BUN (Blood urea nitrogen) | mg/dl | Uricase, Colorimrtry, Enzyme |
| CREA (Creatinine) | mg/dl | Jaffe |
| GLU (Glucose) | mg/dl | HK-G$_6$PD, UV |
| T-CHO (Total cholesterol) | mg/dl | Enzymatic, colorimetry |
| A/G (Albumin globulin ratio) | ratio | ALB/(TP-ALB) |
| TP (Total protein) | g/dl | Biuret |
| ALB (Albumin) | g/dl | BCG |
| CPK (Creatine phosphokinase) | IU/l | UV-Rate |
| TG (Triglyceride) | mg/dl | Lipase, GK, GPO, POD without Glycerol blank |
| T-BIL (Total bilirubin) | mg/dl | Bilirubin oxidase |

performed by comparing the different dose groups with the vehicle control group using Statistical Analysis Systems (SAS/STAT Version 8.1, Cary, NC, USA). The level of statistical significance was set to 5% ($p < 0.05$) and 1% ($p < 0.01$). The descriptive results were presented as mean ± SD.

## RESULTS

***Clinical findings.*** As shown in Table 2, we found some clinical signs, soft feces, liquid feces and vomiting in cynomolgus monkeys treated with inorganic or organic arsenics. Especially, the monkeys in sodium arsenite treated group showed a significant increase in vomiting frequency compared with the monkeys in three organic arsenic-treated groups.

***Hematological parameters.*** The items measured and analytical methods are summarized in Table 3 and the values for hemoglobin (Hb), hematocrit (HCT), reticulocytes, red blood cells (RBC) count, white blood cell (WBC) count, platelet count, mean corpuscular volume (MCV), mean corpuscular hemoglobin (MCH), and mean corpuscular hemoglobin concentration (MCHC) are listed in Table 3. On day 2, they did not show any changes in the parameters after animals with inorganic and organic arsenics. On day 4, the monkeys in inorganic arsenic-treated group showed a decrease in platelet in high dose group, and an increase in monocyte percentage compared with those of control group. On day 8, the monkeys in arsenocholine-treated group showed an increase in reticulocyte percentage in low or high dose group.

***Serum biochemical parameters.*** Table 4 is the list of serum concentrations of aspartate aminotransferase (AST), alanine aminotransferase (ALT), alkaline phosphate(ALP), blood urea nitrogen (BUN), creatinine (CREA), glucose (GLU), total cholesterol (T-CHO), albumin/globulin (A/G), total protein (TP), albumin (ALB), creatine phosphokinase (CPK), triglyceride (TG), total bilirubin (T-BIL). Inorganic group showed decreases in AST and ALT values in low and high dose group compared with those of control group. Arsenobetaine group showed a decrease in AST value and an increase in T-CHO value in low or high dose group.

## DISCUSSION

Exposure to inorganic arsenics in human has been associated with a variety of effects on health, including increases in cardiovascular disease, neurological defects, and neoplasias of the skin, liver, kidney, and bladder (Vega *et al.*, 2001). However, the lack of appropriate animal models and adequate dose-response studies has hampered an understanding of the mechanisms underlying arsenic toxicity. Methylarsonic acid (MMA) and dimethylarsinic acid (DMA) are the main metabolites in man exposed to arsenite (As III) or arsenate (As V), (Crecelius, 1977; Tam *et al.*, 1979; Buchet *et al.*, 1981), while in most mammalian species, DMA is the only methylated metabolite (Charbonneau *et al.*, 1979; Bertolero *et al.*, 1981; Vahter, 1981). The methylated metabolites are readily excreted in the urine (Buchet *et al.*, 1981; Vahter *et al.*, 1984), and have therefore been considered as detoxification products. There is growing evidence that the methylated arsenic compounds, DMA, in particular, cause serious toxicological problems such as DNA damage and chromosomal aberrations (Brown *et al.*, 1997; Yamanaka *et al.*, 1989). The marmoset monkey has been shown to be unable to methylate arsenite, whereas the cynomolgus and rhesus monkeys can methylate it (Vahter *et al.*, 1982; Wildfang *et al.*, 2001; Vahter, 2000). In the present study using cynomolgus monkeys, DMA was found in the urine of the cynomolgus monkeys treated with the arsenite sodium

**Table 2.** Clinical findings of monkeys

| | Test article | | | | | | | | | | | | | | | |
|---|---|---|---|---|---|---|---|---|---|---|---|---|---|---|---|---|
| | Sodium arsenite | | | | Arsenobetaine | | | | Arsenocholine bromide | | | | DMA | | | |
| | Low dose | | High dose | | Low dose | | High dose | | Low dose | | High dose | | Low dose | | High dose | |
| Dose volume (mg/kg) | 2.60 | | 26.01 | | 3.56 | | 71.30 | | 4.90 | | 98.10 | | 4.28 | | 82.70 | |
| Normal | 3[a] | 100[b] | 3 | 100 | 3 | 100 | 3 | 100 | 3 | 100 | 3 | 100 | 3 | 100 | 3 | 100 |
| Soft feces | 1 | 33 | 1 | 33 | 0 | 0 | 0 | 0 | 1 | 33 | 1 | 33 | 0 | 0 | 2 | 66 |
| Liquid feces | 0 | 0 | 1 | 33 | 0 | 0 | 0 | 0 | 0 | 0 | 0 | 0 | 0 | 0 | 0 | 0 |
| Vomiting | 0 | 0 | 3** | 100 | 0 | 0 | 0 | 0 | 1 | 33 | 0 | 0 | 0 | 0 | 0 | 0 |

[a]No. of animals affected, [b]Percent of animals with observation during interval.
Inorganic group (sodium arsenite) (n = 3) vs. organic (n = 9) groups in test articles: $**p < 0.01$ (Statistical Analysis: Fisher's exact Test).

**Table 3.** Comparison of hematological parameters with inorganic arsenic and organic arsenics

(on day 2 after dosing)

| Items | Control (-D6) | Sodium Arsenite | | Arsenobetaine | | Arsenocholine | | DMA | |
|---|---|---|---|---|---|---|---|---|---|
| | | Low | High | Low | High | Low | High | Low | High |
| RBC ($10^6$/μl) | 5.71 ± 0.47 | 6.04 ± 0.74 | 5.93 ± 0.16 | 5.75 ± 0.25 | 5.75 ± 0.21 | 5.25 ± 0.27 | 5.41 ± 0.51 | 5.50 ± 0.20 | 5.80 ± 0.11 |
| HGB (g/dl) | 13.5 ± 0.86 | 14.1 ± 1.26 | 13.9 ± 0.91 | 13.5 ± 0.75 | 13.5 ± 0.61 | 13.1 ± 0.44 | 13.5 ± 0.67 | 12.8 ± 0.58 | 13.5 ± 0.25 |
| HCT (%) | 44.5 ± 3.18 | 45.6 ± 5.47 | 45.1 ± 1.20 | 43.7 ± 3.34 | 44.2 ± 0.46 | 42.2 ± 1.74 | 44.4 ± 2.65 | 42.4 ± 2.04 | 44.3 ± 1.00 |
| MCV(fl) | 78.0 ± 4.05 | 75.6 ± 0.38 | 76.1 ± 1.07 | 75.8 ± 2.90 | 77.0 ± 3.12 | 80.7 ± 7.64 | 82.3 ± 5.53 | 77.1 ± 3.56 | 76.5 ± 2.95 |
| MCH(pg) | 23.7 ± 1.28 | 23.3 ± 1.31 | 23.5 ± 1.10 | 23.5 ± 0.35 | 23.5 ± 1.00 | 25.0 ± 2.16 | 25.1 ± 1.93 | 23.2 ± 0.85 | 23.3 ± 0.79 |
| MCHC (g/dl) | 30.4 ± 0.81 | 30.9 ± 1.81 | 30.9 ± 1.88 | 31.0 ± 0.95 | 30.6 ± 1.14 | 31.0 ± 0.25 | 30.5 ± 0.38 | 30.1 ± 0.26 | 30.5 ± 0.15 |
| PLT ($10^3$/μl) | 386 ± 60.86 | 385 ± 46.6 | 307 ± 39.6 | 403 ± 31.7 | 329 ± 27.1 | 398 ± 96.4 | 333 ± 107.4 | 448 ± 61.2 | 396 ± 50.6 |
| RET (%) | 1.3 ± 0.51 | 1.1 ± 0.40 | 1.5 ± 0.20 | 1.0 ± 0.10 | 1.4 ± 0.06 | 1.2 ± 0.42 | 1.5 ± 0.15 | 0.9 ± 0.25 | 1.3 ± 0.50 |
| WBC($10^3$/μl) | 10.65 ± 2.80 | 13.34 ± 8.28 | 11.99 ± 3.84 | 13.95 ± 2.52 | 13.91 ± 2.29 | 10.18 ± 0.60 | 11.69 ± 1.98 | 9.03 ± 0.73 | 10.32 ± 2.68 |
| NEU (%) | 37.8 ± 10.01 | 50.2 ± 20.34 | 45.4 ± 12.00 | 48.5 ± 22.08 | 47.4 ± 2.85 | 37.8 ± 6.82 | 45.6 ± 3.50 | 40.1 ± 2.12 | 43.2 ± 7.16 |
| LYM (%) | 56.9 ± 10.17 | 43.7 ± 17.90 | 47.2 ± 9.34 | 47.7 ± 21.45 | 47.7 ± 3.03 | 57.6 ± 7.31 | 50.2 ± 4.51 | 53.8 ± 2.87 | 51.3 ± 8.20 |
| MON (%) | 2.9 ± 0.99 | 4.5 ± 2.79 | 4.8 ± 1.93 | 2.5 ± 0.65 | 3.1 ± 1.25 | 2.9 ± 0.98 | 2.9 ± 1.01 | 3.8 ± 1.40 | 4.3 ± 1.17 |
| EOS (%) | 1.6 ± 1.43 | 0.7 ± 0.74 | 1.3 ± 1.68 | 0.6 ± 0.25 | 1.0 ± 0.64 | 1.0 ± 0.57 | 0.7 ± 0.45 | 1.3 ± 1.36 | 0.5 ± 0.36 |
| BAS (%) | 0.2 ± 0.09 | 0.2 ± 0.10 | 0.3 ± 0.06 | 0.2 ± 0.10 | 0.2 ± 0.00 | 0.2 ± 0.00 | 0.2 ± 0.06 | 0.3 ± 0.17 | 0.2 ± 0.10 |
| LUC (%) | 0.7 ± 0.24 | 0.8 ± 0.55 | 1.0 ± 0.75 | 0.4 ± 0.10 | 0.6 ± 0.31 | 0.6 ± 0.17 | 0.4 ± 0.12 | 0.7 ± 0.10 | 0.6 ± 0.20 |

(on day 4 after dosing)

| Items | Control (-D6) | Sodium Arsenite | | Arsenobetaine | | Arsenocholine | | DMA | |
|---|---|---|---|---|---|---|---|---|---|
| | | Low | High | Low | High | Low | High | Low | High |
| RBC ($10^6$/μl) | 5.71 ± 0.5 | 5.6 ± 0.2 | 5.4 ± 0.2 | 5.6 ± 0.3 | 5.4 ± 0.2 | 5.5 ± 0.6 | 5.0 ± 0.3 | 5.5 ± 0.3 | 5.2 ± 0.3 |
| HGB (g/dl) | 13.5 ± 0.9 | 13.0 ± 0.1 | 12.8 ± 0.6 | 13.2 ± 0.8 | 12.7 ± 0.3 | 13.6 ± 0.5 | 12.6 ± 0.5 | 12.8 ± 1.0 | 12.1 ± 0.7 |
| HCT (%) | 44.5 ± 3.2 | 43.1 ± 1.6 | 41.9 ± 1.1 | 43.1 ± 1.9 | 41.7 ± 0.6 | 45.0 ± 2.4 | 41.1 ± 0.3 | 43.3 ± 4.1 | 39.7 ± 2.6 |
| MCV(fl) | 78.0 ±4.1 | 76.6 ± 1.4 | 77.1 ± 0.9 | 76.9 ± 3.8 | 77.6 ± 3.6 | 81.8 ± 4.9 | 82.1 ± 5.4 | 78.4 ± 5.7 | 77.1 ± 4.0 |
| MCH(pg) | 23.7 ±1.3 | 23.2 ± 0.9 | 23.6 ± 0.8 | 23.5 ± 0.5 | 23.7 ± 0.7 | 24.7 ± 1.9 | 25.2 ± 2.3 | 23.1 ± 1.0 | 23.5 ± 0.9 |
| MCHC (g/dl) | 30.4 ±0.8 | 30.3 ± 1.4 | 30.7 ± 1.3 | 30.6 ± 1.1 | 30.5 ± 0.7 | 30.2 ± 0.6 | 30.6 ± 1.0 | 29.6 ± 0.9 | 30.4 ± 0.4 |
| PLT ($10^3$/μl) | 386.0 ±60.9 | 383.0 ± 10.5 | 290.0 ± 36.1* | 377.0 ± 56.9 | 322.0 ± 41.0 | 391.0 ± 123.6 | 321.0 ± 116.3 | 459.0 ± 46.0 | 373.0 ± 52.1 |
| RET (%) | 1.3 ± 0.5 | 1.2 ± 0.3 | 1.6 ± 0.2 | 1.6 ± 0.1 | 1.5 ± 0.1 | 1.6 ± 0.3 | 1.6 ± 0.2 | 1.3 ± 0.4 | 1.3 ± 0.5 |
| WBC($10^3$/μl) | 10.65 ± 2.8 | 10.6 ± 2.5 | 10.6 ± 3.9 | 12.1 ± 1.0 | 9.57 ± 0.9 | 11.33 ± 0.7 | 9.8 ± 0.4 | 10.73 ± 1.1 | 8.92 ± 0.3 |
| NEU (%) | 37.8 ± 10.0 | 40.0 ± 2.3 | 37.1 ± 8.4 | 44.2 ± 12.4 | 32.8 ± 5.2 | 41.4 ± 1.2 | 38.4 ± 6.2 | 46.6 ± 5.3 | 46.3 ± 5.6 |
| LYM (%) | 56.9 ± 10.2 | 50.5 ± 1.7 | 54.9 ± 7.7 | 52.1 ± 12.2 | 62.4 ± 6.6 | 53.8 ± 2.6 | 56.8 ± 7.3 | 47.8 ± 7.3 | 48.4 ± 7.2 |
| MON (%) | 2.9 ± 1.0 | 5.6 ± 1.0* | 4.7 ± 1.0 | 2.2 ± 1.1 | 2.9 ± 1.1 | 2.8 ± 1.0 | 2.6 ± 0.7 | 3.9 ± 1.8 | 3.9 ± 2.3 |
| EOS (%) | 1.6 ± 1.4 | 2.2 ± 1.9 | 2.0 ± 1.4 | 0.7 ± 0.2 | 1.2 ± 0.7 | 1.0 ± 1.0 | 1.4 ± 0.7 | 0.6 ± 0.5 | 0.6 ± 0.7 |
| BAS (%) | 0.2 ± 0.1 | 0.4 ± 0.1 | 0.3 ± 0.2 | 0.2 ± 0.1 | 0.2 ± 0.1 | 0.3 ± 0.2 | 0.2 ± 0.1 | 0.3 ± 0.1 | 0.1 ± 0.1 |
| LUC (%) | 0.7 ± 0.2 | 1.2 ± 0.5 | 1.1 ± 0.5 | 0.6 ± 0.3 | 0.6 ± 0.1 | 0.7 ± 0.3 | 0.6 ± 0.4 | 0.8 ± 0.1 | 0.6 ± 0.1 |

(on day 8 after dosing)

| Items | Control (-D6) | Sodium arsenite | | Arsenobetaine | | Arsenocholine | | DMA | |
|---|---|---|---|---|---|---|---|---|---|
| | | Low | High | Low | High | Low | High | Low | High |
| RBC ($10^6$/μl) | 5.71 ± 0.47 | 5.66 ± 0.26 | 5.64 ± 0.18 | 5.53 ± 0.27 | 5.65 ± 0.10 | 5.35 ± 0.37 | 5.23 ± 0.42 | 5.48 ± 0.37 | 5.30 ± 0.42 |
| Hb (g/dl) | 13.5 ± 0.86 | 13.0 ± 0.40 | 13.3 ± 0.46 | 13.0 ± 0.81 | 13.4 ± 0.47 | 13.2 ± 0.44 | 13.1 ± 0.58 | 12.7 ± 1.04 | 12.5 ± 1.01 |
| Hct (%) | 44.5 ± 3.18 | 43.8 ± 1.88 | 43.8 ± 1.89 | 42.6 ± 2.79 | 44.4 ± 2.60 | 43.8 ± 1.18 | 43.0 ± 1.97 | 42.7 ± 3.97 | 41.6 ± 3.90 |
| MCV(fl) | 78.0 ± 4.05 | 77.5 ± 1.92 | 77.7 ± 1.07 | 77.0 ± 3.61 | 78.6 ± 4.76 | 82.1 ± 6.45 | 82.4 ± 6.09 | 77.8 ± 3.42 | 78.4 ± 3.62 |
| MCH(pg) | 23.7 ± 1.28 | 23.0 ± 0.98 | 23.6 ± 0.95 | 23.4 ± 0.44 | 23.8 ± 0.53 | 24.7 ± 2.08 | 25.0 ± 1.96 | 23.2 ± 0.99 | 23.5 ± 0.98 |
| MCHC (g/dl) | 30.4 ± 0.81 | 29.7 ± 0.74 | 30.4 ± 1.32 | 30.4 ± 1.15 | 30.3 ± 1.66 | 30.1 ± 0.17 | 30.4 ± 0.17 | 29.8 ± 0.45 | 30.0 ± 0.38 |
| PLT ($10^3$/μl) | 386 ± 60.86 | 427 ± 56.7 | 355 ± 17.9 | 381 ± 59.6 | 337 ± 48.4 | 391 ± 110.5 | 348 ± 105.9 | 471 ± 73.7 | 408 ± 71.0 |
| RET (%) | 1.3 ± 0.51 | 2.0 ± 0.62 | 1.8 ± 0.15 | 1.9 ± 0.49 | 1.7 ± 0.36 | 2.4 ± 0.29* | 2.2 ± 0.40* | 1.6 ± 0.47 | 1.8 ± 0.53 |
| WBC($10^3$/μl) | 10.65 ± 2.80 | 10.48 ± 4.10 | 11.29 ± 5.59 | 9.81 ± 1.97 | 10.17 ± 0.72 | 8.65 ± 0.74 | 10.81 ± 2.85 | 8.84 ± 1.74 | 9.21 ± 1.43 |
| NEU (%) | 37.8 ± 10.01 | 31.8 ± 15.14 | 41.4 ± 17.01 | 31.7 ± 6.76 | 33.9 ± 5.58 | 33.9 ± 4.57 | 49.7 ± 10.91 | 43.6 ± 5.15 | 44.2 ± 3.02 |
| LYM (%) | 56.9 ± 10.17 | 61.4 ± 16.18 | 52.8 ± 18.01 | 64.0 ± 6.85 | 61.6 ± 7.05 | 62.3 ± 4.57 | 45.2 ± 10.35 | 51.9 ± 7.35 | 50.6 ± 4.83 |
| MON (%) | 2.9 ± 0.99 | 3.0 ± 1.01 | 2.6 ± 0.50 | 2.9 ± 1.77 | 2.5 ± 0.52 | 2.0 ± 0.55 | 2.8 ± 1.19 | 3.1 ± 1.68 | 3.6 ± 2.07 |
| EOS (%) | 1.6 ± 1.43 | 2.7 ± 1.86 | 2.2 ± 1.96 | 0.8 ± 0.42 | 0.9 ± 0.56 | 0.9 ± 0.55 | 1.1 ± 0.31 | 0.8 ± 0.60 | 0.7 ± 0.71 |
| BAS (%) | 0.2 ± 0.09 | 0.4 ± 0.21 | 0.3 ± 0.12 | 0.2 ± 0.06 | 0.5 ± 0.42 | 0.2 ± 0.06 | 0.5 ± 0.31 | 0.1 ± 0.06 | 0.3 ± 0.17 |
| LUC (%) | 0.7 ± 0.24 | 0.7 ± 0.40 | 0.7 ± 0.50 | 0.4 ± 0.15 | 0.6 ± 0.20 | 0.6 ± 0.17 | 0.8 ± 0.35 | 0.6 ± 0.06 | 0.6 ± 0.06 |

[a]Mean ± SD; Control group: n = 12, Test Article group: n = 3.
VC vs. low dose and high dose in each test article: $*p < 0.05$.

**Table 4.** Comparison of serum biochemical parameters with inorganic arsenic and organic arsenics (on day 8 after dosing)

| Items | Control (-D6) | Sodium Arsenite | | Arsenobetaine | | Arsenocholine | | DMA | |
|---|---|---|---|---|---|---|---|---|---|
| | | Low | High | Low | High | Low | High | Low | High |
| AST (IU/l) | 50.7 ± 9.01 | 33.5 ± 4.8* | 31.7 ± 5.4* | 38.9 ± 4.0* | 29.4 ± 1.7** | 51.5 ± 11.3 | 41.7 ± 16.5 | 41.6 ± 6.0 | 38.5 ± 5.8 |
| ALT (IU/l) | 70.4 ± 22.00 | 34.1 ± 6.6* | 33.7 ± 4.3* | 57.0 ± 33.7 | 45.7 ± 27.7 | 59.9 ± 0.4 | 53.4 ± 11.1 | 39.1 ± 13.5 | 39.8 ± 14.9 |
| ALP (IU/l) | 1213.2 ± 532.7 | 1277.4 ± 649.0 | 1171.4 ± 599.1 | 1213.6 ± 514.5 | 1179.4 ± 541.2 | 912.5 ± 397.9 | 854.1 ± 334.4 | 1256.0 ± 272.5 | 1042.1 ± 259.6 |
| BUN (mg/dl) | 20.2 ± 8.6 | 17.8 ± 1.0 | 17.0 ± 0.9 | 16.6 ± 1.5 | 16.4 ± 3.5 | 19.0 ± 4.4 | 17.9 ± 4.7 | 16.9 ± 1.8 | 16.7 ± 1.8 |
| CREA (mg/dl) | 1.0 ± 0.2 | 1.1 ± 0.1 | 1.01 ± 0.1 | 1.0 ± 0.0 | 1.0 ± 0.0 | 1.1 ± 0.2 | 1.09 ± 0.2 | 1.1 ± 0.2 | 1.0 ± 0.1 |
| GLU (mg/dl) | 64.6 ± 11.2 | 67.7 ± 19.4 | 58.0 ± 11.0 | 62.0 ± 16.7 | 66.3 ± 14.0 | 63.2 ± 8.7 | 59.9 ± 12.1 | 58.4 ± 7.1 | 54.4 ± 4.6 |
| T-CHO (mg/dl) | 128.6 ± 10.4 | 84.6 ± 11.7 | 87.7 ± 3.1 | 144.8 ± 9.2* | 147.3 ± 15.2** | 115.2 ± 2.6 | 115.7 ± 5.0 | 126.8 ± 16.9 | 126.3 ± 20.53 |
| A/G (ratio) | 1.5 ± 0.3 | 1.4 ± 0.1 | 1.4 ± 0.1 | 1.5 ± 0.2 | 1.5 ± 0.2 | 1.5 ± 0.0 | 1.5 ± 0.0 | 1.4 ± 0.2 | 1.4 ± 0.1 |
| TP (g/dl) | 7.0 ± 1.0 | 7.0 ± 0.5 | 6.8 ± 0.6 | 7.3 ± 0.2 | 6.9 ± 0.1 | 7.2 ± 0.6 | 6.7 ± 0.9 | 7.3 ± 0.1 | 6.9 ± 0.0 |
| ALB (g/dl) | 4.1 ± 0.3 | 4.1 ± 0.4 | 4.0 ± 0.4 | 4.4 ± 0.2 | 4.1 ± 0.2 | 4.3 ± 0.4 | 4.0 ± 0.6 | 4.4 ± 0.2 | 4.0 ± 0.2 |
| CPK (IU/l) | 204.0 ± 63.1 | 144.0 ± 53.1 | 128.0 ± 52.2 | 145.0 ± 45.7 | 137.0 ± 36.5 | 165.0 ± 21.0 | 122.0 ± 30.4 | 126.0 ± 47.5 | 113.0 ± 50.5 |
| TG (mg/dl) | 51.0 ± 32.4 | 21.8 ± 3.37 | 24.7 ± 3.33 | 31.7 ± 10.0 | 31.8 ± 17.6 | 22.0 ± 9.1 | 20.5 ± 10.6 | 23.9 ± 4.9 | 20.4 ± 4.7 |
| T-BIL (mg/dl) | 0.2 ± 0.1 | 0.2 ± 0.0 | 0.2 ± 0.0 | 0.2 ± 0.0 | 0.2 ± 0.5 | 0.2 ± 0.0 | 0.1 ± 0.0 | 0.2 ± 0.0 | 0.1 ± 0.0 |

[a]Mean ± SD; Control group: n = 12, Test Article group: n = 3.
VC vs. low dose and high dose in each test article: *$p < 0.05$, **$p < 0.01$.

(data was not shown).

Until the 1950s, organic arsenic compounds such as salvarsan or neosalvarsan were used to treat syphilis, and patients treated with these compounds occasionally developed a rash known as postsalvarsan or postneosalvarsan exanthema (Uede and Furukawa, 2003). A recent study of acute arsenic poisoning showed gastrointestinal disorders such as nausea, abdominal pain, diarrhea, and vomiting including skin manifestations and cardiovascular and neurological disorders in human (Uede and Furukawa, 2003). In the present study, we also found soft and liquid feces, and vomiting in all treated groups with inorganic and organic arsenics. This finding agreed with the gastrointestinal disorders of previous report (Uede and Furukawa, 2003). Especially, inorganic arsenic (sodium arsenite) treated group showed a significant increase in vomiting frequency compared with three organic arsenic groups (Table 2). This result indicates that inorganic arsenic is more toxic than three organic arsenics. Organic arsenics have relatively low toxicity compared to inorganic arsenics (e.g., arsenate and arsenite forms) (Mandal and Suzuki, 2002; Vahter, 2000).

In the present study, inorganic arsenic-treatedgroup showed a decrease in platelet counts and an increase in monocyte value on day 4 and arsenocholine-treated group showed an increase in reticulocyte percentage on day 8. Inorganic arsenic-treated group also showed decreases in AST and ALT values, whereas arsenobetaine-treated group showed a decrease in AST and an increase in T-CHO. However, these changes were within the physiological ranges (Kim *et al.*, 2005), showing that the single dose of inorganic and organic arsenics did not affect at least hematological and serum biochemical parameters in this study.

In the present study, we tried to screen the toxic effects of organic arsenics (arsenobetaine and arsenocholine), a final product of arsenic methylation (DMA), and inorganic arsenic (sodium arsenite) on hematological and serum biochemical parameters for pursuing the toxicity study of the repeated dose of arsenics in cynomolgus monkeys. Further studies are required to identify chemical metabolites after administration of various types of arsenic and to determine the relationship between methylation and detoxification in organic and inorganic arsenics. The present study with a single dose of arsenics will provide valuable indicators to plan the study of toxicity using the repeated dose of arsenics incynomolgus monkeys.

## ACKNOWLEDGEMENTS

This study was supported by KFDA (No. 08152-432; 2007-2008). The technical assistance provided by Pil-Soo Lee, Kap-Soo Lee, Nam-Soo Park and Ku-Hwa Kang is gratefully acknowledged.
